# dadi.CUDA: Accelerating Population Genetics Inference with Graphics Processing Units

**DOI:** 10.1093/molbev/msaa305

**Published:** 2021-01-22

**Authors:** Ryan N Gutenkunst

**Affiliations:** Department of Molecular and Cellular Biology, University of Arizona, Tucson, AZ, USA

**Keywords:** population genetics, demographic history, GPU computing, dadi

## Abstract

dadi is a popular but computationally intensive program for inferring models of demographic history and natural selection from population genetic data. I show that running dadi on a Graphics Processing Unit can dramatically speed computation compared with the CPU implementation, with minimal user burden. Motivated by this speed increase, I also extended dadi to four- and five-population models. This functionality is available in dadi version 2.1.0, https://bitbucket.org/gutenkunstlab/dadi/.

Population genetic data contain information about the history of the sampled populations, but extracting that information often demands computationally expensive modeling. dadi is widely used for inferring models of demographic history (Gutenkunst et al. 2009) and natural selection (Kim et al. 2017) from data summarized by an allele frequency spectrum (AFS). The user specifies a model with parameters for population sizes, migration rates, divergence times, and/or selection coefficients. Given a set of parameters, dadi computes the expected AFS and the composite likelihood of the data. The parameters are optimized to maximize that likelihood, and AFS computation dominates optimization run time. Here, I show that Graphics Processing Units (GPUs) can massively speed AFS computation and thus overall inference.

GPUs have rarely been applied to population genetic simulation or inference. Lawrie (2017) developed a GPU implementation of the single-locus Wright–Fisher model, finding speedups of over 250 times compared with a CPU implementation. Zhou et al. (2015) implemented a subset of the IM program for inferring demographic models (Hey and Nielsen 2004) on a GPU, finding speedups of around 50 times.

For dadi, the core computations are solving a partial differential equation (PDE) for the population distribution of allele frequencies ϕ and integrating over that distribution to compute the AFS. Solving the PDE reduces to solving a large number of tridiagonal linear systems (fig. 1*A*). To solve these systems on the GPU, I used the Valero-Lara et al. (2018) algorithm, as implemented in the Nvidia Compute Unified Device Architecture (CUDA) library. dadi is primarily written in Python; to interface with CUDA I used the PyCUDA (Klöckner et al. 2012) and scikit-cuda (Givon et al. 2019) libraries. Computing the AFS from ϕ reduces to matrix multiplication, which did not consistently benefit from a GPU, so this step remains on the CPU.

**Fig. 1.**
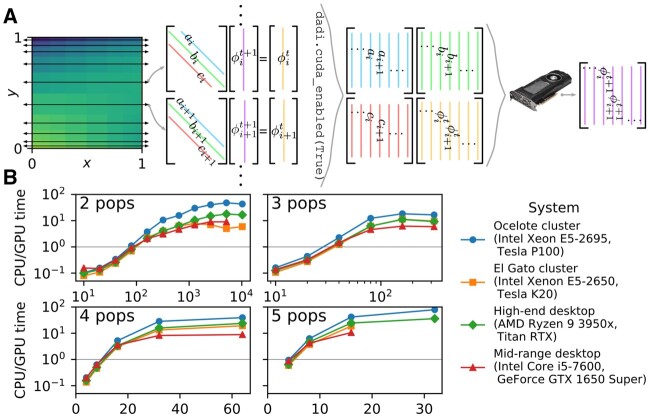
(*A*) Illustration of dadi integration. During each timestep, the population allele density ϕ is updated for each population axis. Each row and column over which ϕ is approximated yields a tridiagonal linear system. In the GPU implementation, these systems are solved in parallel. (*B*) Ratios of CPU to GPU times to compute the AFS for several models on several computing systems, versus AFS size. Absolute computation times are shown in supplementary figure S1, Supplementary Material online. The largest AFS size tested on each system was constrained by GPU memory.

For the end user, dadi GPU usage requires only a single call to dadi.cuda_enabled(True).

I evaluated performance by comparing times to compute the AFS for models from stdpopsim (Adrion et al. 2020) on several computing systems. To achieve reasonable accuracy, for spectra with *n* chromosomes per population, I used extrapolation grid points of (⌊1.1*n*⌋ + 2, ⌊1.2*n*⌋ + 4, ⌊1.3*n*⌋ + 6). The benchmarking code is available in the dadi source distribution.

Historically, dadi is most often used with two- or three-population models. I thus tested the two-population model of Li and Stephan (2006) and the three-population model of Gutenkunst et al. (2009). Using a GPU was beneficial if the sample size was greater than 70 for two populations and 30 for three populations (fig. 1*B* and supplementary fig. S1, Supplementary Material online); such values are common in data analyses.

Given the GPU speedup, I extended dadi to four- and five-population models. Tests with the four-population New World model from Gutenkunst et al. (2009) and the five-population archaic admixture model from Ragsdale and Gravel (2019) again showed substantial GPU benefits (fig. 1*B* and supplementary fig. S1, Supplementary Material online). Models with more populations typically have more free parameters, which increases the expected number of optimization steps. So optimizing the parameters of four- and five-population models may be challenging even with GPU acceleration.

The ultimate benefit of GPU computing is high performance at low cost. At time of writing, the consumer GeForce GPU costs less than $200, but it substantially speeds computation. The data center Tesla P100 costs roughly $6,000, and it can be used to calculate larger spectra because of its larger memory. The Valero-Lara et al. (2018) algorithm is typically bound not by computation, but rather by memory bandwidth within the GPU. Compared with consumer GPUs, data center GPUs typically have a large advantage in double-precision computation but a more modest advantage in memory bandwidth. So for dadi usage, consumer GPUs may provide better performance versus cost.

The GPU speed improvements increase dadi’s competitiveness with other methods for calculating the expected AFS, such as moments (Jouganous et al. 2017). The speed differences between the GPU and CPU implementations of dadi are larger than the differences between the CPU implementations of dadi and moments, in both present (supplementary fig. S2, Supplementary Material online) and previous (Jouganous et al. 2017) benchmarks. Other factors may influence choice between these methods, including level of support and access to more advanced features, such linkage disequilibrium statistics in moments (Ragsdale and Gravel 2019) or distributions of fitness effects in dadi (Kim et al. 2017).

## Supplementary Material

Supplementary data are available at *Molecular Biology and Evolution* online.

## Supplementary Material

msaa305_Supplementary_Data

## References

[msaa305-B1] Adrion JR , Cole CB, Dukler N, Galloway JG, Gladstein AL, Gower G, Kyriazis CC, Ragsdale AP, Tsambos G, Baumdicker F, et al 2020. A community-maintained standard library of population genetic models. eLife 9:e54967. 32573438 10.7554/eLife.54967 PMC7438115

[msaa305-B2] Givon LE , Unterthiner T, Erichson NB, Chiang DW, Larson E, Pfister L, Dieleman S, Lee GR, van der Walt S, Menn B, et al 2019. scikit-cuda 0.5.3: a Python interface to GPU-powered libraries. Available from: 10.5281/zenodo.3229433.

[msaa305-B3] Gutenkunst RN , Hernandez RD, Williamson SH, Bustamante CD. 2009. Inferring the joint demographic history of multiple populations from multidimensional SNP frequency data. PLoS Genet. 5(10):e1000695. 19851460 10.1371/journal.pgen.1000695 PMC2760211

[msaa305-B4] Hey J , Nielsen R. 2004. Multilocus methods for estimating population sizes, migration rates and divergence time, with applications to the divergence of *Drosophila pseudoobscura* and *D. persimilis*. Genetics 167(2):747–760. 15238526 10.1534/genetics.103.024182 PMC1470901

[msaa305-B5] Jouganous J , Long W, Ragsdale AP, Gravel S. 2017. Inferring the joint demographic history of multiple populations: beyond the diffusion approximation. Genetics 206(3):1549–1567. 28495960 10.1534/genetics.117.200493 PMC5500150

[msaa305-B6] Kim BY , Huber CD, Lohmueller KE. 2017. Inference of the distribution of selection coefficients for new nonsynonymous mutations using large samples. Genetics 206(1):345–361. 28249985 10.1534/genetics.116.197145 PMC5419480

[msaa305-B7] Klöckner A , Pinto N, Lee Y, Catanzaro B, Ivanov P, Fasih A. 2012. PyCUDA and PyOpenCL: a scripting-based approach to GPU run-time code generation. Parallel Comput. 38(3):157–174.

[msaa305-B8] Lawrie DS. 2017. Accelerating Wright-Fisher forward simulations on the graphics processing unit. G3 (Bethesda) 7(9):3229–3236. 28768689 10.1534/g3.117.300103 PMC5592947

[msaa305-B9] Li H , Stephan W. 2006. Inferring the demographic history and rate of adaptive substitution in Drosophila. PLoS Genet. 2(10):1580–1589. 10.1371/journal.pgen.0020166 PMC1599771 17040129

[msaa305-B10] Ragsdale AP , Gravel S. 2019. Models of archaic admixture and recent history from two-locus statistics. PLoS Genet. 15(6):e1008204. 31181058 10.1371/journal.pgen.1008204 PMC6586359

[msaa305-B11] Valero-Lara P , Martínez-Pérez I, Sirvent R, Martorell X, Peña AJ. 2018. cuThomasBatch and cuThomasVBatch, CUDA routines to compute batch of tridiagonal systems on NVIDIA GPUs. Concurrency Computat Pract Exper. 30(24):e4909.

[msaa305-B12] Zhou C , Lang X, Wang Y, Zhu C. 2015. gPGA: GPU accelerated population genetics analyses. PLoS One 10(8):e0135028. 26248314 10.1371/journal.pone.0135028 PMC4527771

