# Supplementary material for: dadi.CUDA: Accelerating Population Genetics Inference with Graphics Processing Units
Source: Mol Biol Evol. 2021 Jan 22;38(5):2177–8. doi: 10.1093/molbev/msaa305 (PMC8097298; doi:10.1093/molbev/msaa305)
Supplement: msaa305_Supplementary_Data [file msaa305_supplementary_data.pdf]

Supplemental Information

dadi.CUDA: Accelerating population genetics inference with Graphics Processing Units

Ryan N. Gutenkunst

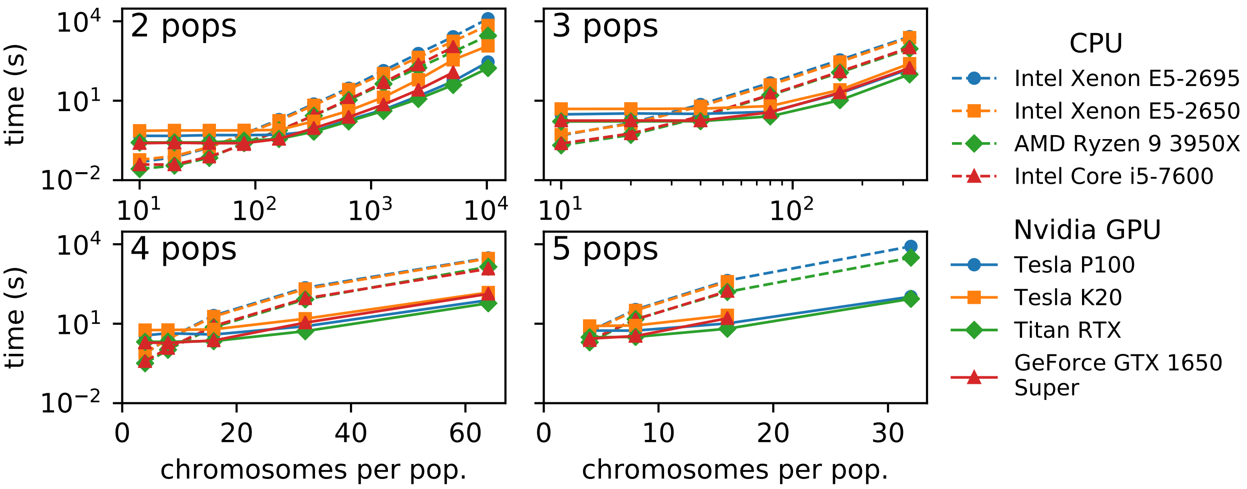

Figure S1: Run times for CPU and GPU dadi implementations, for the same models and systems as Fig. 1B.

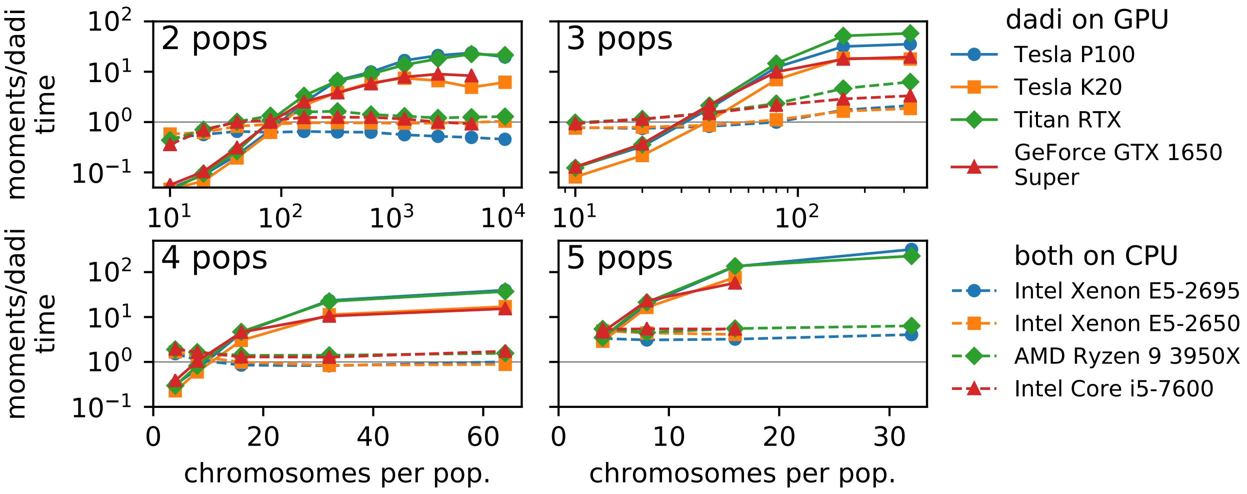

Figure S2: Ratios of moments to dadi times to compute the AFS. dadi results are the same as in Fig. 1B. moments results were calculated with a timestep factor of 0.05.
